# The First Case Report of Ectopic Hepatic Tissue in a Guinea Pig

**DOI:** 10.1155/2021/6626299

**Published:** 2021-01-23

**Authors:** Tohru Kimura, Kengo Inaka

**Affiliations:** Laboratory Animal Science, Joint Faculty of Veterinary Medicine, Yamaguchi University, 1677-1, Yoshida, Yamaguchi 753-8515, Japan

## Abstract

Ectopic hepatic tissue is an extremely rare developmental abnormality in human beings. Although this lesion is likewise rare in veterinary medicine and there were sparse reports in several species such as dogs, cats, cows, and calves, we incidentally encountered ectopic hepatic tissue in a guinea pig. In the case of guinea pigs, we report for the first time the occurrence of ectopic hepatic tissue implanted in the gallbladder. The healthy guinea pig remained asymptomatic, and its macroscopic findings also showed no abnormalities. Hematological examinations exhibited moderate decreases in white cell counts, hemoglobin concentrations, and packed cell volume ratio. Serum biochemical examinations showed decreases in total protein and albumin and increases in glucose levels, alkaline phosphatase, lactate dehydrogenase, creatine kinase, and *γ*-glutamyl transpeptidase. There were slight changes in electrolytes (Cl, Ca, and Mg) and inorganic phosphorus, indicating minor deviations from physiologic ranges. An increase in TBIL concentrations was not found in this examination. Histopathological examination revealed the presence of normal hepatic structures (hepatocytes and hepatic cords) within the wall of the normal gallbladder. The vascular and bile duct structures and portal triads were not observed in the ectopic hepatic tissue. In conclusion, this microectopic hepatic tissue in a guinea pig was characterized by the embedded structures of hepatic tissues, without foreign body reaction.

## 1. Introduction

Ectopic hepatic tissue can be attached to the liver or completely independent from the liver [1]. Ectopic hepatic tissue (hepatic choristoma) means abnormal development of hepatic tissues in an anatomical location different from their physiological site. Ectopic hepatic tissue bears no definite relation to the original liver. Ectopic hepatic tissue is an extremely rare developmental abnormality that is found in abdominal and thoracic sites of human beings [1]. This spontaneous abnormality has been only rarely described in the field of veterinary medicine [2–9], including a case of ectopic liver and gallbladder in an artificial cloned dog [10]. The aim of the current work was to report a hepatic choristoma incidentally encountered in a guinea pig used as a control animal in another experiment. Although we did not observe macroscopic changes in the gallbladder and liver or clinicopathological alterations, histopathological examination revealed ectopic hepatic tissue in the guinea pig. To the best of the author's knowledge, in the case of guinea pigs, we report for the first time the occurrence of ectopic hepatic tissue implanted in the gallbladder.

## 2. Case Presentation

### 2.1. Materials and Methods

Female 8-week-old genetically unmodified Hartley strain guinea pigs served as a control group in another experiment. In the control animals, we have incidentally encountered a case of ectopic hepatic tissue implanted in the gallbladder. This guinea pig was not accompanied by any clinical signs.

Female Hartley albino guinea pigs were primarily introduced at 3 weeks of age (Kyudo Co., Ltd., Saga, Japan). After 1 week of acclimatization, animals that were healthy and 450-500 g in body weight were selected for our other study. The animals were housed in groups of 2 head in cages (CL-0143 (R-2), W 355 × D 499 × H 198 mm, Crea Japan Inc., Tokyo, Japan) kept at a room temperature of 24 ± 2°C, a relative humidity of 50 ± 5%, and an air exchange rate of 15 times/hour. The room was artificially illuminated for 12 hrs./12 hrs. light-dark cycle daily (light 07 : 00-19 : 00, dark 19 : 00-07 : 00). The animals had free access to water bottles and a solid diet (CG-7, Crea Japan Inc., Tokyo, Japan).

Under systemic anesthesia with isoflurane, blood samples were collected from the caudal vena cava of the guinea pig using no anticoagulant. At 30 minutes after collection of blood samples, sera were separated by centrifugation at 1,500 g for 10 minutes for biochemical analysis. For hematological samples, blood was collected into tubes containing K_2_EDTA.

In hematological examinations, the following parameters were investigated using an automated cell counter (Microsemi LC-662 Horiba Co. Ltd., Kyoto, Japan): white cell count (WBC), red blood cell count (RBC), hemoglobin concentration (Hb), packed cell volume (PCV) ratio, mean corpuscular volume (MCV), mean corpuscular hemoglobin (MCH), mean corpuscular hemoglobin concentration (MCHC), red blood cell distribution width (RDW), platelet count (PLT), mean platelet volume (MPV), Plateletcrit (PCT), and platelet distribution width (PDW).

In serum biochemical examinations, the following parameters were measured using a blood chemistry analyzer (Dry Chem NX 500V: Fuji Film Co. Ltd., Tokyo, Japan): total protein (TP), albumin (Alb), globulin (Glob), albumin : globulin (A/G) ratio, total bilirubin (TBil), urate (UA), blood urea nitrogen (BUN), creatinine (CRE), glucose (GLU), triglycerides (TG), total cholesterol (TCHO), aspartate aminotransferase (AST), alanine aminotransferase (ALT), alkaline phosphatase (ALP), lactate dehydrogenase (LDH), cholinesterase (ChE), leucine aminopeptidase (LAP), creatine kinase (CK), *γ*-glutamyl transpeptidase (GGT), amylase (AMS), electrolytes (Na, K, Cl, Ca), inorganic phosphorus (IP), and magnesium (Mg).

The guinea pig was euthanized by carbon dioxide exposure. Immediately after euthanasia, the guinea pig was necropsied, and tissue samples were taken for the histopathological examinations. The tissue specimens were fixed in 10% neutral buffered formalin, and 4 *μ*m paraffin sections were stained with hematoxylin and eosin (HE). We examined multiple slide to verify whether the choristoma was attached to the “mother” liver or not.

All procedures involving animals were approved by the Institutional Animal Care and Use Committee of Yamaguchi University and followed the Guidelines of Animal Care and Experiments of Yamaguchi University. The animal care and use program for Advanced Research Center for Laboratory Animal Science in Yamaguchi University have been accredited by AAALAC International since 2018.

## 3. Results

The healthy control guinea pig remained asymptomatic, and then, its macroscopic findings also showed no abnormalities at autopsy. Hematological and serum biochemical profiles are shown in Tables 1 and 2. In hematological examinations, WBCs, Hb concentrations, and PCV ratio moderately decreased as compared with reference values [11]. Serum biochemical examinations showed decreases in TP and Alb and increases in GLU levels, ALP, LDH, CK, and GGT. There were slight changes in electrolytes (Cl, Ca, IP, and Mg), indicating minor deviations from physiologic ranges reported in the literature [12]. An increase in TBIL concentrations was not found in this examination.

**Figure 1:**
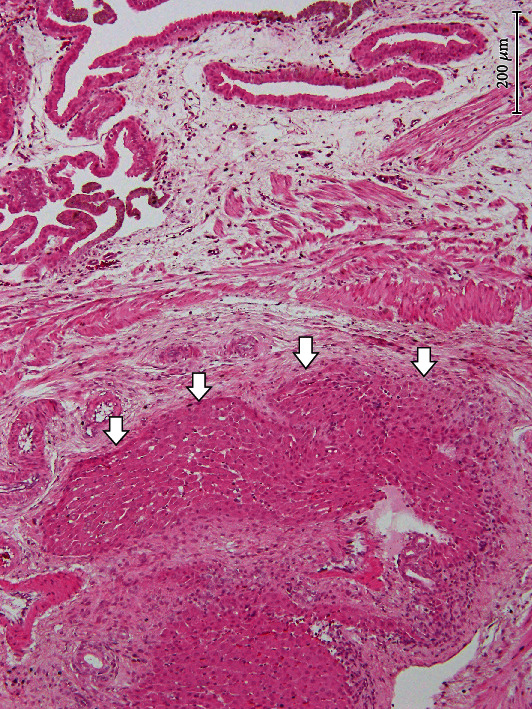
Microscopic finding of ectopic hepatic tissue. Microectopic hepatic tissue (arrowheads) was found beneath the lamina propria mucosae of the normal gallbladder, intermingled with the muscular fibers of the muscularis. HE stain. ×100, bar = 200 *μ*m.

Histopathological examination revealed the presence of a hepatic choristoma without morphological features attributable to hepatocellular carcinoma. As shown in Figure 1, the ectopic hepatic tissue was located within the wall of the gallbladder beneath the lamina propria mucosae, intermingled with the muscular fibers of the muscularis. Hepatocytes in the ectopic hepatic tissue were morphologically normal hepatic cells frequently arranged in hepatic cords. However, arrangement of hepatic cords in structure attributable to hepatic lobules with central veins was not found in the investigated lesions. In addition, the ectopic hepatic tissue was accompanied by the biliary drainage system (i.e., ductules and ducts) and the hepatic triads (i.e., the association of an interlobular artery, an interlobular vein, and an interlobular bile duct). We did not find foreign body reactions in this lesion, such as hepatocytes surrounded by fibrous capsule and infiltration of giant cells.

## 4. Discussion

Ectopic hepatic tissue has been known to occur due to an uncommon failure of embryological development [13]. The incidence of this lesion in human beings has been described as anywhere from 0.05% (3/5,500) to 0.28% (3/1,060) [14–16]. According to laparoscopic surgical observations, its incidence has been estimated to range from 0 (0/2,650) [17] to 0.7% (14/1,802) [18]. Although some cases of human patients develop epigastric pain, abdominal acute pain, or biliary colic [16, 19–21], this disease is clinically silent and is usually identified during abdominal surgery.

In human clinical cases of ectopic hepatic tissue, serum biochemical examinations provided some diagnostic information such as notably elevated AMS, GGT, and ALT activities [21, 22]. Hepatocellular carcinoma in a young dog represented the following high serum liver enzymes: AST (55 U/L), ALT (124 U/L), ALP (2321 U/L), and GGT (420 I/U) [23]. In hematological and serum biochemical examinations, our guinea pig did not show marked changes as compared with human and canine patients. The present examinations provided no evidence of severe hepatocellular damage and biliary obstruction. It was probable that microectopic hepatic tissue caused no clinical abnormalities in the guinea pig, indicating a physically healthy individual. The aforementioned increase in hepatic enzyme activity in human and canine cases resulted from the complications correlated with the presence of heterotopic tissue in the gallbladder, such as obstacle to bile outflow.

Ectopic hepatic tissue is divided into 4 distinct categories: (1) ectopic hepatic tissue that is not connected to the main liver and is usually attached to the gallbladder or intra-abdominal ligaments wall, (2) microscopic ectopic liver found occasionally in the gallbladder wall, (3) a large accessary liver attached to the “mother” liver by a stalk, and (4) a small accessory liver lobe attached to the main liver [1]. The present case in the guinea pig corresponded to the lesion of category 2. In both the humans and guinea pigs, microectopic hepatic tissue was prone to occur beneath the lamina propria mucosae of the gallbladder.

It was important that ectopic hepatic tissue did not contained a complete hepatic lobular architecture lacking a complete vascular and ductal system. This hepatic tissue might be functionally impaired by the lack of vascular supply and biliary drainage, as pointed out in a previous report [22]. A previous study described that histopathological findings of calves with ectopic liver were hemorrhage, local area of coagulative necrosis, and diffuse fatty changes. Fat cyst besides normal central vein was found and swollen hepatocytes with granular cytoplasm besides Kupffer cell hyperplasia in the portal area and within the hepatic parenchyma. Proliferation of fibrous connective tissue in the portal area and formation of new bile ductules were also detected [8]. In feline case, the mass had histopathological characteristics of normal hepatic tissue [7]. An intrathoracic liver with pleural effusion in a dog histopathologically showed that the mass was composed of normal hepatic parenchyma [3]. Our microectopic hepatic tissue was characterized by the embedded structures of hepatic tissues, without foreign body reaction. These results differed from abovementioned histopathological findings in several species.

According to a recent study on ectopic liver and gallbladder in a cloned dog [10], this disorder is possibly a nonheritable anomaly, and this lesion may be the result of an aberrant epigenetic modification during embryogenesis. Because nonfunctional microectopic hepatic tissue has no deleterious effects on the gallbladder, we consider that it is rare to find such a lesion in guinea pigs. It was possible that most of microectopic hepatic tissue (microscopic ectopic liver) was early absorbed into the surrounding tissue.

We concluded that the present case report provided the first information regarding ectopic hepatic tissue in a guinea pig. The ectopic hepatic tissue was located within the wall of the gallbladder beneath the lamina propria mucosae, and this nonfunctional tissue showed no foreign body reaction.

## Figures and Tables

**Table 1:** Hematological profiles.

Parameters	Values	Reference values (female guinea pigs: mean ± SD or range) [11]
WBC (×10^9^/L)	4	7.04 ± 2.01
RBC (×10^12^/L)	4.57	5.19 ± 0.40
Hb (g/L)	118	133 ± 8
PCV ratio	0.38	0.429 ± 0.03
MCV (fL)	82.2	82.9 ± 3.9
MCH (pg)	25.8	25.7 ± 0.8
MCHC (g/L)	313	303 ± 9
RDW (%)	12	―
PLT (×10^9^/L)	571	250-850
MPV (fL)	5.5	―
PCT (%)	0.315	―
PDW (%)	11.9	―

**Table 2:** Serum biochemical profiles.

Parameters	Values	Reference values (female guinea pigs: mean ± SD) [12]
TP (g/dL)	39	48 ± 3.4
Alb (g/dL)	18	24.2 ± 1.4
Glob (g/dL)	21	17.1 ± 1.6
A/G ratio	0.86	―
TBIL (*μ*mol/L)	1.71	5.47 ± 1.20
UA (*μ*mol/L)	95.2	55.32 ± 13.68
BUN (mmol/L)	5.2	7.68 ± 2.08
CRE (*μ*mol/L)	0.38	0.124 ± 0.031
GLU (mmol/L)	9.10	4.94 ± 0.53
TG (mmol/L)	0.71	0.49 ± 0.30
TCHO (mmol/L)	0.75	0.69 ± 0.29
AST (U/L)	35	45.50 ± 7.00
ALT (U/L)	24	38.80 ± 7.15
ALP (U/L)	365	65.80 ± 5.46
LDH (U/L)	179	52.1 ± 11.2
ChE (U/L)	10	―
LAP (U/L)	518	―
CK (U/L)	362	110 ± 20
GGT (U/L)	46	10 ± 3
AMS (U/L)	1611	―
Na (mmol/L)	128	125 ± 0.96
K (mmol/L)	4.1	5.06 ± 0.93
Cl (mmol/L)	91	96.50 ± 1.19
Ca (mmol/L)	2.13	2.67 ± 0.15
IP (mmol/L)	2.58	1.71 ± 0.36
Mg (mmol/L)	1.52	1.01 ± 0.11

## Data Availability

No data were used to support this study.

## References

[1] Collan Y., Hakkiluoto H. J. (1978). Ectopic liver. *Annales Chirurgiae et Gynaecologiae*.

[2] Burton I. R., Limpus K., Thompson K. G., Owen M. C., Worth A. J. (2005). Ectopic hepatocellular carcinoma in a dog. *New Zealand Veterinary Journal*.

[3] Iwaki Y., Takagi S., Morishita K., Hanazono K., Hosoya K., Okumura M. (2017). An intrathoracic ectopic liver with pleural effusion in a dog. *Jap. J. Vet. Res*.

[4] Lande R., Dvorak L., Gardiner D. W., Bahr A. (2015). Ectopic intrathoracic hepatic tissue and accessory lung lobe aplasia in a dog. *Journal of the American Animal Hospital Association*.

[5] Dhaliwal R. S., Lacey J. K. (2009). Ectopic hepatic parenchyma attached to the diaphragm: simulating a pulmonary mass in a cat. *Journal of the American Animal Hospital Association*.

[6] Jones B. R., Alley M. R., Cribb S. B. (1986). Pericardial ectopic liver in a cat. *New Zealand Veterinary Journal*.

[7] França T. N., Nogueira V. A., Alves L., Brito M. F., Peixoto P. V. (2010). Intrapericardial hepatic choristoma in a cat – a case report. *Revista Brasileira de Medicina Veterinária*.

[8] Gomaa M., Gouda S. M. (2015). Ectopic liver anomaly in umbilical region of newly born calves: clinical, pathological, sonographical and surgical investigations. *Global Vet*.

[9] Hifumi T., Kawaguchi H., Yamada M., Miyoshi N. (2014). Intrathoracic ectopic liver in a cow. *The Journal of Veterinary Medical Science*.

[10] Kim M. J., Kang S. C., Kim J. H. (2015). Ectopic liver and gallbladder in a cloned dog: possible nonheritable anomaly. *Theriogenology*.

[11] Zimmerman K. L., Moore D. M., Smith S. A., Weiss D. J., Wardrop K. J. (2010). *Hematology of the guinea pig*.

[12] Sharp P., Kurtz D. M., Travlos G. S. (2018). *The Laboratory Guinea Pig*.

[13] Martinez C. A. R., de Resende H. C., Rodrigues M. R., Sato D. T., Brunialti C. V., Palma R. T. (2013). Gallbladder-associated ectopic liver: a rare finding during a laparoscopic cholecystectomy. *International Journal of Surgery Case Reports*.

[14] Eiserth P. (1941). Beiträge zur Kenntnis der Nebenlebern. *Virchows Archiv für Pathologische Anatomie und Physiologie und für Klinische Medizin*.

[15] Watanabe M., Matsura T., Takatori Y. (1989). Five cases of ectopic liver and a case of accessory lobe of the liver. *Endoscopy*.

[16] Avdaj A., Namani S., Cake A., Bytyqi A. (2020). Case report of ectopic hepatic tissue, a rare finding during a laparoscopic cholecystectomy. *International Journal of Surgery Case Reports*.

[17] Orlando R., Lirussi F. (2000). Congenital anomalies of the liver: laparoscopic observations. *Gastrointestinal Endoscopy*.

[18] Sato S., Watanabe M., Nagasawa S., Niigaki M., Sakai S., Akagi S. (1998). Laparoscopic observations of congenital anomalies of the liver. *Gastrointestinal Endoscopy*.

[19] Lundy J., Johnson E., Edwards K., Rivera D. (2005). Laparoscopic management of gallbladder-associated ectopic liver. *J. Soc. Laparoendosc. Surg*.

[20] Termos S., AlDuwaisan A., Alali M., Faour H., AlHomoud H., Alsaleh A. (2017). Gallbladder-associated symptomatic hepatic choristoma: should you resect?. *International Journal of Surgery Case Reports*.

[21] Burke E. P., Harkins P., Arih I., O’Donoghue G. (2018). A case of ectopic liver tissue adherent to the gallbladder. *Journal of Surgical Case Reports*.

[22] Arakawa M., Kimura Y., Sakata K., Kubo Y., Fukushima T., Okuda K. (1999). Propensity of ectopic liver to hepatocarcinogenesis: case reports and a review of the literature. *Hepatology*.

[23] Teshima T., Matsumoto H., Shigihara K. (2013). Hepatocellular carcinoma in a young dog. *The Canadian Veterinary Journal*.

